# Multi-Omics Analysis of the Prognosis and Biological Function for TRPV Channel Family in Clear Cell Renal Cell Carcinoma

**DOI:** 10.3389/fimmu.2022.872170

**Published:** 2022-04-26

**Authors:** Yuxiong Jiang, Dongxu Han, Yifan Zhao, Chen Zhang, Xiujuan Shi, Wenyu Gu

**Affiliations:** ^1^Department of Urology, Shanghai Tenth People’s Hospital, School of Medicine, Tongji University, Shanghai, China; ^2^School of Medicine, Tongji University, Shanghai, China; ^3^Department of Hematology, Mianyang Central Hospital, Mianyang, China

**Keywords:** TRPV, ccRCC, prognosis, immune infiltration, TCGA

## Abstract

**Background:**

The transient receptor potential vanilloid (TRPV) channels family, TRPV1-6, has been identified to profoundly affect a wide spectrum of pathological processes in various cancers. However, the biological function and prognostic value of TRPVs in clear cell renal cell carcinoma (ccRCC) are still largely unknown.

**Methods:**

We obtained the gene expression data and clinical information of 539 ccRCC patients from The Cancer Genome Atlas (TCGA) database. A series of databases were used for data processing and visualization, including GEPIA, GeneMANIA, MethSurv, GSCA, TIMER, and starBase databases.

**Results:**

The mRNA expression of TRPV2/3 was upregulated while the expression of TRPV5/6 was downregulated in ccRCC tumor tissues. TRPV family members in ccRCC were rarely mutated (nearly 7 frequencies). The ROC curve showed that TRPV2/5/6 had a high diagnostic ability in discriminating ccRCC from the control samples (AUC>0.9). Higher levels of TRPV3 expression were associated with poor prognosis of ccRCC patients, while higher expression of TRPV4 was associated with favorable prognosis. The expression of TRPV3 in normal and ccRCC tissues was validated by Immunohistochemistry, and its expression was remarkably related to high histologic grade and advanced stage. Besides, TRPV3 exhibit a reduction of DNA methylation level with tumor progression, and 12 CpGs of TRPV3 were associated with a significant prognosis. In addition, TRPV3 expression was significantly associated with the accumulation of several tumor-infiltrating immune cells, especially regulatory T cells. Furthermore, high levels of TRPV3 induced the expression of immune checkpoints such as LAG3, CTLA4, PDCD1, and TIGIT. Finally, we predicted a key SNHG3/AL513497.1-miR-10b-5p-TRPV3 axis linking to carcinogenesis and progression of ccRCC.

**Conclusion:**

Our study may uncover TRPV channels–associated molecular mechanisms involved in the tumorigenesis and progression of ccRCC. TRPV family members might be diagnosed and prognostic markers and potential therapeutic targets for ccRCC patients.

## Introduction

Renal cell carcinoma (RCC) is one of the most lethal malignant tumors of the urinary system. Clear cell renal cell carcinoma (ccRCC) is the most prevalent histologic type, accounting for approximately 75% of RCC (1, 2). At present, the most effective treatment for ccRCC is surgery, but due to ccRCC not being susceptible to adjuvant chemotherapy and radiotherapy, relapse and metastasis following surgery are common (3). Recently, immune checkpoint inhibitors (ICIs) have demonstrated efficacy in patients with metastatic ccRCC (4). However, the prognosis of ccRCC patients is still poor, and the current morbidity and mortality rates remain high (5). Therefore, revealing the underlying molecular mechanisms of ccRCC and screening effective prognostic biomarkers is crucial for prolonging the survival of ccRCC patients.

Increasing evidence indicates that calcium ions and their channels play an important role in tumor cell proliferation, invasion, migration, and tumor angiogenesis (6). In the process of tumor cell migration, cell morphology and adhesion characteristics change repeatedly with the fluctuation of cytoplasmic calcium concentration (7). In addition, tumor cells can promote proliferation, enhance invasion, and escape apoptosis by reducing calcium influx or down-regulating the permeability of calcium channels (8). Transient receptor potential (TRP) channels are a major class of non-selective cation channels, which are involved in sensing various intracellular and extracellular stimuli (9). TRP channels share common structural characteristics, including six transmembrane domains S1-S6, C and N terminus in cells, and a pore-forming ring between S5 and S6 transmembrane segments (10). The transient receptor potential vanilloid (TRPV) family is one of the subfamilies of TRP channels and consists of six members: TRPV1 (NM_018727.5), TRPV2 (NM_016113.5), TRPV3 (NM_145068.4), TRPV4 (NM_021625.5), TRPV5 (NM_019841.7), and TRPV6 (NM_018646.6) (11). Numerous studies have demonstrated that the TRPV family was involved in tumorigenesis and progression for a wide variety of tumors (12–14).

A previous study reported that TRPV5/6 expression was remarkably decreased in human renal cell carcinoma compared with normal kidney tissues (15). In addition, vitamin D receptor overexpression could inhibit proliferation, migration, and invasion of renal cell carcinoma cells by increasing TRPV5 expression (16). Moreover, low expression of TRPV1 was found to be significantly associated with multiple key molecules of the classical pathways involved in the origin, progression, and tumor microenvironment of ccRCC, including MET, VHL, HIF1A, MTOR, MAPK1, TP53, CTNNB1 (17). However, the diagnostic and prognostic values of different TRPV members in ccRCC have not been investigated, and the association of TRPV family with ccRCC microenvironment is still not determined. In the present study, we comprehensively analyzed the prognosis and biological significance for the TRPV family in ccRCC based on multi-omics data. Firstly, we identified the expression difference of TRPV members between ccRCC tumor and normal tissues. Next, we evaluated diagnostic and prognostic values of TRPV members in ccRCC patients. After that, we assessed the association between TRPV3 expression and the levels of tumor-infiltrating immune cells (TIICs) and immune checkpoints expression. Finally, we established a potential lncRNA -miRNA- mRNA network linked to carcinogenesis and progression of ccRCC. Our findings may be helpful to amplify the molecular mechanisms in the pathophysiological process of ccRCC and provide insights for exploring novel biomarkers for clinical diagnosis, prognosis, and immunotherapy targets for ccRCC patients.

## Materials and Methods

### Data Acquisition

We downloaded gene expression data (HTSeq-FPKM) of 539 ccRCC cases and 72 control cases from the TCGA database (https://portal.gdc.cancer.gov/). The corresponding clinical information of ccRCC patients was also obtained, including gender, pathologic stage, TNM stage, histologic stage, serum calcium, and overall survival (OS) event. Two microarray datasets, GSE53757 and GSE73731 datasets, were also downloaded from the GEO database. The GSE53757 datasets consists of 72 ccRCC tissues and matched normal kidney tissues (18), and the GSE73731 datasets contains 265 ccRCC tissues (19). The platform file of microarray datasets is GPL570 [HG-U133_Plus_2]. The “Limma” package of R software (x64 3.2.1) was used to rectify and normalize the raw data.

### mRNA Expression Analysis

Firstly, we used the module of “GTEx Expression” in the GSCA database (20) to observe the mRNA expression of TRPV1-6 in multiple human tissues from healthy individuals. Besides, the interactive body maps of TRPV1-6 were constructed by GEPIA database (21), which visualized the median expression of TRPV1-6 in normal tissues and organs. On the basis of GSCA database, we further compared the expression difference of TRPV1-6 between normal and 14 tumor tissues, involving THCA, KIRP, BLCA, LIHC, HNSC, BRCA, LUAD, PRAD, ESCA, KICH, LUSC, ccRCC, STAD, COAD. The expression of TRPVs in ccRCC was compared in unpaired samples (539 tumors vs 72 control groups) and paired tumor samples (72 tumors vs 72 control groups) based on TCGA-KIEC cohort. Statistics were calculated by Wilcoxon rank sum test and paired t-tests, respectively. Furthermore, GSE53757 datasets was employed to verify the above results. P-value < 0.05 was considered to be significant.

### Genetic Alterations and Interaction Network of TRPV Family

The alterations of TRPV family members in ccRCC was detected in 538 ccRCC samples from Firehose Legacy project of TCGA through cBioPortal database (22). The web platform also provided the association between TRPVs mutation and overall survival of ccRCC patients. Next, we generated the gene network of TRPV family and predicted the potential function that they may contribute to by GeneMANIA database (23). Moreover, the STRING database was introduced to develop a protein-protein interaction network of TRPV family. The main parameters were as follows: organism (“Homo sapiens”), network type (“full STRING network”), minimum required interaction score (“medium confidence 0.400”). The correlation among TRPV family members was evaluated by Spearman correlation analysis based on TCGA data.

### Prognostic and Diagnostic Value Analysis

The “Survival” R package was utilized to organize the survival information of ccRCC samples and the Cox regression model was performed to selected the prognosis biomarkers from TRPV family members. The Kaplan-Meier curve was used to compare the overall survival of ccRCC patients between TRPV3/4 high expression and TRPV3/4 low-expression groups. Logistic regression was conducted to analyze the relationship between TRPV3 expression and clinical variables. We also constructed a nomogram model to predict the 1-, 3-,5-year overall survival of ccRCC patients by “rms” R package. The “pROC” R package was used to reflect the efficacy of TRPV members in the diagnosis of ccRCC. P-value < 0.05 was considered to be significant. The area under the curve (AUC) of receiver operating characteristic (ROC) curve could summarize the diagnostic effect.

### DNA Methylation Analysis

The relationship between TRPV3 mRNA expression and DNA methylation levels was evaluated through “Methylation & expression” module in the GSCA database by Spearman correlation analysis. Given that there are several methylation sites in one gene, the database calculated the site most negatively associate with TRPV3 expression. The UALCAN database (24) was applied to compare the promoter methylation levels of TRPV3 between normal control tissues and ccRCC tissues. The methylation levels of TRPV3 in ccRCC patients grouped by different clinicopathological features also detected, including nodal metastasis status, tumor grade and individual cancer stages. The level of DNA methylation was measured by the Beta value, which ranges from 0 (unmethylated) to 1 (fully methylated). P-value < 0.05 was considered to be significant. The methylation map of TRPV3 in ccRCC was downloaded from “Gene visualization” module of MethSurv database (25). We also explored the influence of DNA methylation of each CpGs in TRPV3 on the multivariable survival of ccRCC patients using “Single CpG” module of MethSurv database.

### Function Enrichment Analysis

According to the median of TRPV3 expression, ccRCC patients were divided into TRPV3-high groups and TRPV3-low groups based on TCGA-KIRC cohort. The “DESeq2” R package was used to identify the differently expressed genes (DEGs) between TRPV3-high and TRPV3-low groups. Thresholds for the classification of DEGs were up-regulated: log2 fold change values ≥ 1.5 or down-regulated: log2 fold change ≤ −1.5. P < 0.05 was used as the cutoff criteria. Next, we performed Kyoto Encyclopedia of Genes and Genomes (KEGG) and Gene Ontology (GO) enrichment analysis to explore the underlying functional annotation of these TRPV3-related DGEs in ccRCC through “ClusterProfiler” R package.

### Immune-Related Analysis

The CIBERSOFT algorithm was utilized to calculate the proportion of 22 immune cells in each sample based on GSE53757 datasets. The difference of tumor-infiltrating immune cells (TIICs) between TRPV3-high and TRPV3-low groups was further compared through “violet” R package. Furthermore, the “reshape2” R package was applied to explore the correlation of TRPV3 expression with the levels of TIICs. We also determined the impact of the abundance of regulatory T cells (Tregs) on the cumulative survival of ccRCC patients in TRPV3-high and TRPV3-low groups through the “Outcome” module in the TIMER database (26). The expression correlation between TRPV3 and immune checkpoints was evaluated using the “ggplot2” R software package and visualized as co expression heat map and scatter map.

### Prediction of a LncRNA-miRNA-mRNA Axis in ccRCC

We predicted the upstream regulatory network of miRNAs-TRPV3 through “miRNA Regulation” module in GSCA database, which included multiple prediction programs, including papers, mir2disease, TarBase, miRTarBase, miRanda, and targetscan database. The expression association between paired miRNA and mRNA was tested by “miRNA-Target CoExpression” module analysis in starBase database (27). We also obtained upstream potential LncRNAs of candidate miRNAs from “miRNA-lncRNA” module in starBase database. The correlation of LncRNAs with candidate miRNAs and TRPV3 expression were subsequently evaluated through Spearman correlation analysis based on TCGA-KIRC data. The expression and survival analysis of miRNAs and LncRNAs were also performed on the basis of TCGA-KIRC data. P-value < 0.05 was considered to be significant.

### Immunohistochemistry (IHC) Staining

This study was approved by the Institutional Research Ethics Committee of Tongji University. Written informed consent was obtained from all the participants. Six cancerous and adjacent noncancerous tissues were collected and fixed in 10% formalin for 1 week and then embedded in paraffin. Four-micrometer sections of tissue specimens were prepared and deparaffinized and antigen was retrieved by microwaving. Immunostaining was performed with following TRPV3 monoclonal antibodies: (Abcam, ab85022).

## Results

### Gene Expression of TRPV Family Members

**Figure 1:**
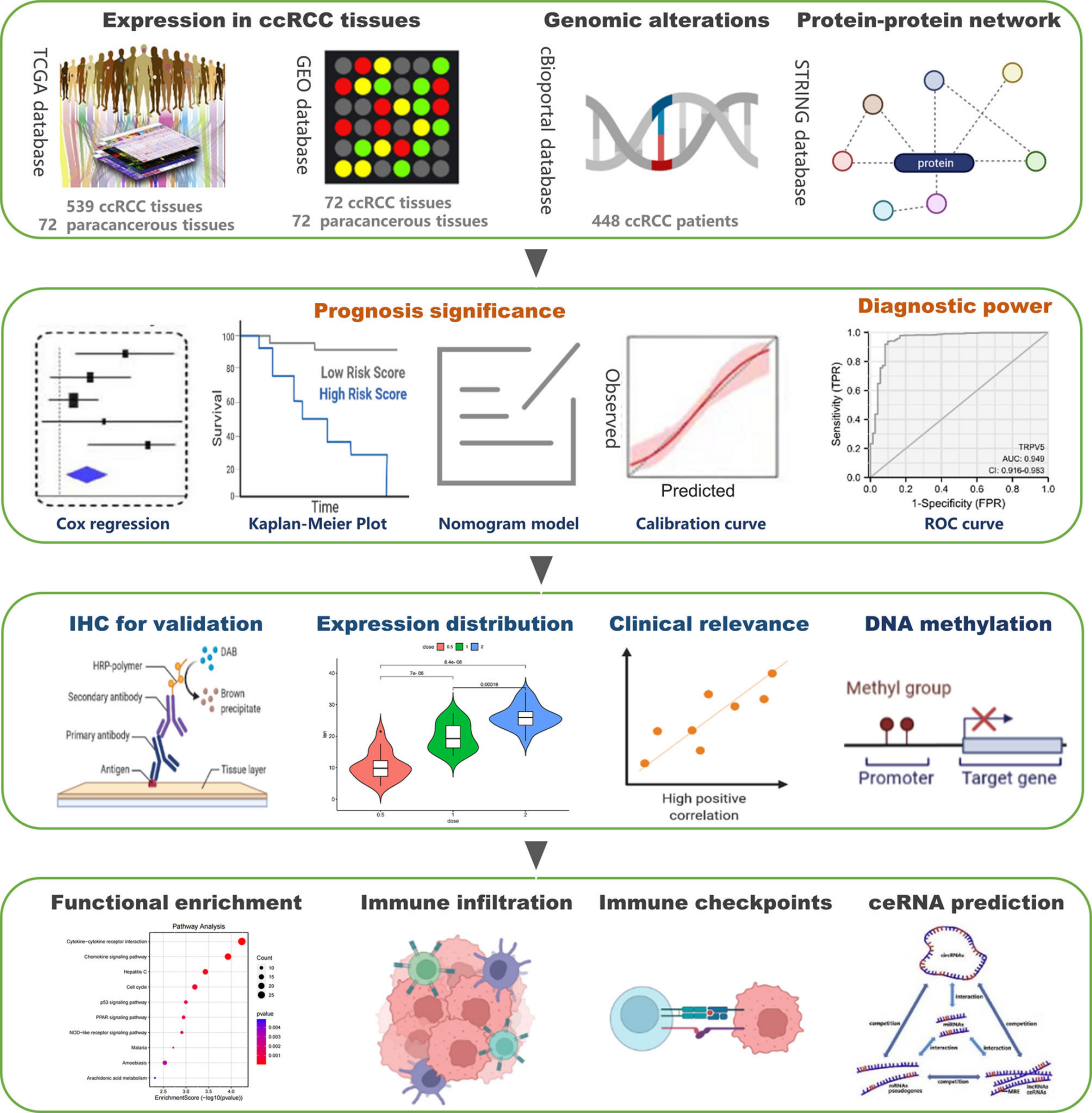
The schematic diagram of analysis.

**Figure 2:**
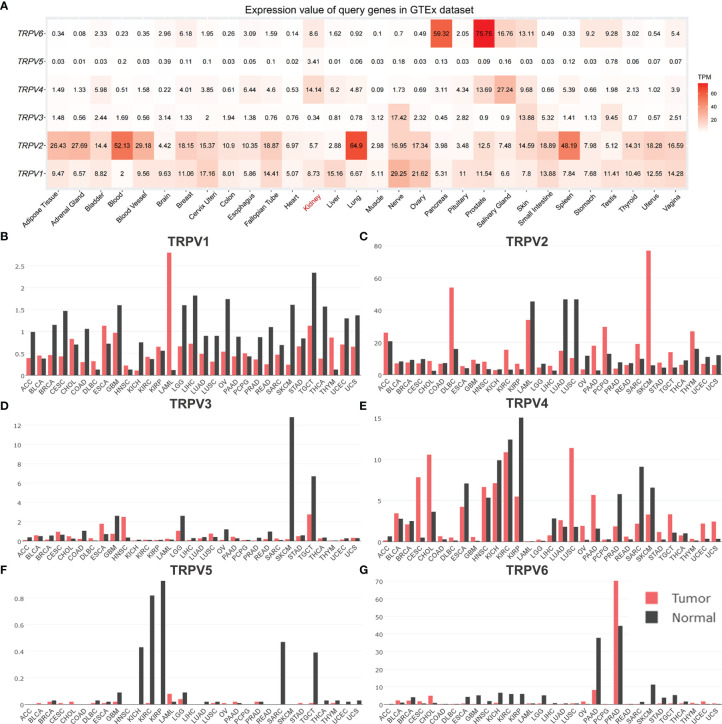
The distribution of TRPV family in human normal and tumor tissues. **(A)** Heatmap of the expression profiles of TRPV members in the GTEx dataset. The median expression of **(B)**TRPV1, **(C)** TRPV2, **(D)** TRPV3, **(E)** TRPV4, **(F)** TRPV5 **(G)** TRPV6 in normal tissues (black) and tumors (red) in various organs. Bar plot constructed on the basis of the GEPIA database.

**Figure 3:**
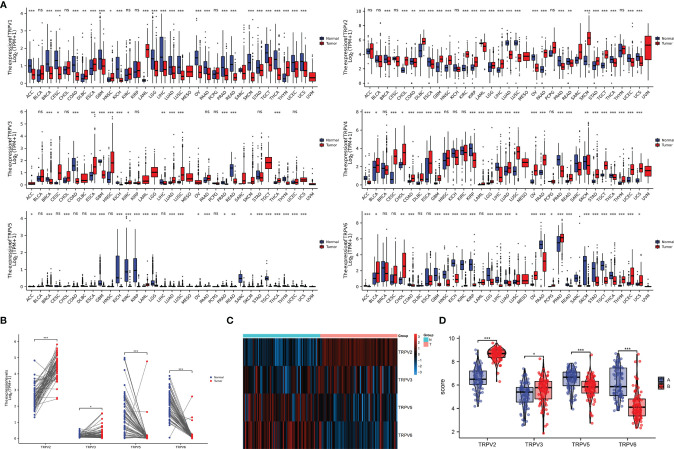
The mRNA expression of TRPV family in ccRCC tissues. **(A)** The expressing levels of TRPV1-6 across diverse cancer types. **(B)** Analysis of TRPV2/3/5/6 expression in 72 pairs of ccRCC and adjacent normal tissues.*P<0.05; P **<0.01; ***P<0.001; ns, no significance.**(C)** The heatmap of TRPV2/3/5/6 expression in GSE53757 datasets. **(D)** Analysis of TRPV2/3/5/6 expression in ccRCC and adjacent normal tissues based on GSE53757 datasets. *P<0.05; ***P<0.001.

In order to comprehensively analyze the function of TRPV family in humans, we first explored the distribution of TRPV1-6 in human normal organs and tissues based on the GTEx database (**Figure 1**). As displayed in **Figure 2A**, the expression of TRPV2 in the blood, lung, and spleen, the expression of TRPV6 in pancreas and the prostate were significantly upregulated. In healthy kidney tissues, the expression of TRPV3 was obviously lower than other family members. These results suggest that the expression of TRPV family members is tissue-specific. Next, we obtained the gene expression profile of TRPV1-6 across tumor and paired normal tissues from GEPIA database. The height of bar plot represents the median expression of TRPVs in tissues. As a result, TRPV family members exhibited different expression patterns between tumors and normal tissues (**Figures 2B–G**). Among 31 types of human tumors, TRPV1-6 were abnormally expressed in 25, 24, 14, 25, 23, 25 tumors, respectively (**Figure 3A**). Then, we used unpaired samples (539 tumors vs 72 control groups) and 72 paired tumor/non-tumor samples from TCGA-KIRC database to explore the expression difference of TRPVs between ccRCC and normal tissues. The results showed that TRPV2 and TRPV3 were upregulated in ccRCC tumor tissues, whereas TRPV5 and TRPV6 were downregulated in tumor tissues (**Figure 3B**). The same results were obtained in GSE53757 dataset, which consisted of 72 ccRCC tissues and matched normal kidney tissues (**Figures 3C, D**).

### Genetic Alterations of TRPV Family and Gene and Protein Network

**Figure 4:**
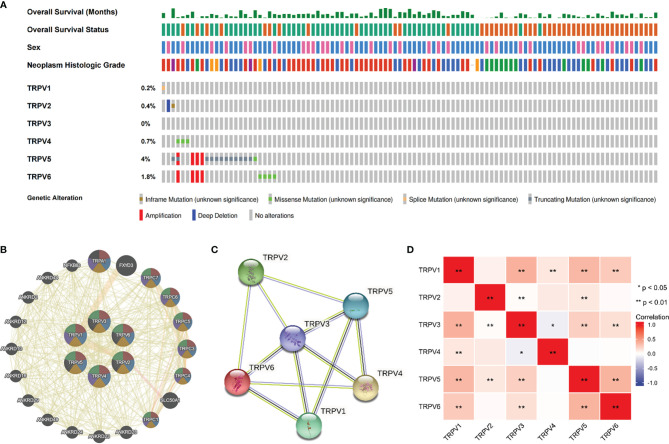
Genomic alterations of TRPV family members and gene–gene and protein–protein interaction network of genes of the TRPV family. **(A)** The cBioPortal database provides an overview of genomic alterations of the TRPV family members in ccRCC patients. **(B)** The gene network associated with the TRPV family analyzed by GeneMANIA database. **(C)** A network diagram of interactions between proteins encoded by genes of the TRPV family. **(D)** The correlation analysis among TRPV family members. *P<0.05; **P<0.01.

We then employed the cBioportal tool to assess the frequencies of TRPV members alterations in 538 KIRC samples. As shown in **Figure 4A**, TRPV family were rarely mutated (TRPV1, 0.2%; TRPV 2, 0.4%; TRPV3, 0%; TRPV4, 0.7%; TRPV5, 4%; TRPV6, 1.8%), which was highly conserved. Besides, genomic alterations of TRPV family had no influence on the overall survival (OS) of ccRCC patients (**Figure S1**). The gene-gene network performed by GeneMANIA database showed that TRPV family interacted with 20 potential target genes (**Figure 4B**). After that, we constructed the protein-protein interacted (PPI) network to evaluate the correlation among TRPV family through STRING website. The results demonstrated that TRPV family members had a close connection (PPI enrichment p-value:2.71E-13). In addition, we noticed that TRPV3 was located in the center of the network and might be the most critical member of the TRPV family (**Figure 4C**). Next, the correlation among TRPV family in mRNA levels was evaluated using TCGA data. There was a significant positive correlation among most TRPV family members, while TRPV3 had a negative correlation with TRPV4 (**Figure 4D**).

### Survival Analysis of TRPV Family in ccRCC

**Figure 5:**
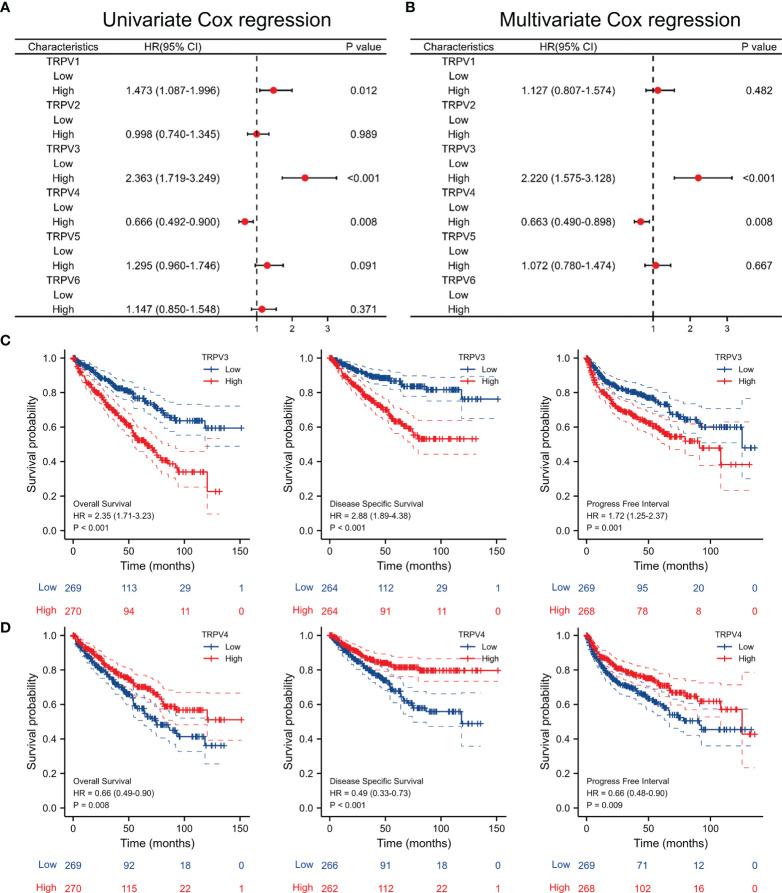
Survival analysis of TRPV family in ccRCC. **(A)** Univariate Cox regression analyses of TRPV1-6 in ccRCC. **(B)** Multivariate Cox regression analyses of TRPV1-6 in ccRCC. **(C)** Kaplan-Meier survival curves comparing the high and low expression of TRPV3 in ccRCC patients, including overall survival, disease specific survival and progress free interval survival. **(D)** Kaplan-Meier survival curves comparing the high and low expression of TRPV4 in ccRCC patients, including overall survival, disease specific survival and progress free interval survival.

Univariate Cox regression analysis revealed that high expression of TRPV1 and TRPV3 while low expression of TRPV4 associated with poor prognosis of ccRCC patients (**Figure 5A**). At Multivariate Cox regression analysis, TRPV3 high expression and TRPV4 low expression were independent risk factors for OS in ccRCC patients (**Figure 5B**). Kaplan-Meier analysis certified that ccRCC patients with high TRPV3 expression suffered shorter survival, including overall survival, disease specific survival, and progress free interval survival (**Figure 5C**). However, high TRPV4 expression predicted a favorable prognosis (**Figure 5D**). We further investigated the impact of TRPV3 and TRPV4 expression on ccRCC prognosis in different subgroups. As shown in **Table 1**, high expression of TRPV3 remarkably affected the OS in ccRCC cases of Stage1/2/4, female and male, Grade2/3, and mutation burden high/low. For TRPV4, high mRNA expression was associated with prolonged survival among Stage1/4, female and male, Grade2/3/4, high mutation burden (**Table 1**). These results suggest that TRPV3 and TRPV4 could serve as biomarkers for poor and good prognosis of ccRCC patients, respectively.

**Table 1:** Correlation of TRPV3 and TRPV4 mRNA expression and clinical prognosis in ccRCC patients with different clinicopathological factors by Kaplan-Meier plotter.

Clinicopathological characteristics	N	Overall survival (530)			
		TRPV3		TRPV4	
		Hazard ratio	p-value	Hazard ratio	p-value
**Stage**					
1	265	2.74 (1.51-4.98)	**0.00054**	0.53 (0.28-0.97)	**0.038**
2	57	3.1 (0.85-11.28)	0.071	0.38 (0.13-1.14)	0.072
3	123	2.72 (1.5-4.92)	**0.00062**	0.55 (0.3-1.02)	0.053
4	82	2.05 (1.25-3.38)	**0.0039**	0.45 (0.26-0.77)	**0.003**
**Gender**					
female	186	3.27 (1.94-5.57)	**2.70E-06**	0.45 (0.27-0.75)	**0.0017**
male	344	2.9 (1.99-4.23)	**8.40E-09**	0.51 (0.34-0.75)	**0.00061**
**Grade**					
1	\			\	
2	227	3.01 (1.66-5.46)	**0.00014**	0.48 (0.27-0.88)	**0.015**
3	206	2.87 (1.79-4.59)	**4.40E-06**	0.46 (0.29-0.74)	**0.00088**
4	75	2.04 (1.03-4.01)	**0.036**	0.57 (0.31-1.04)	0.063
**Mutation burden**					
high	168	2.8 (1.61-4.86)	**0.00014**	0.39 (0.22-0.67)	**0.00044**
low	164	4.23 (1.58-11.32)	**0.0018**	0.66 (0.3-1.46)	0.3

Bolded values are significant at p < 0.05.

### Prognostic and Diagnosis Significance of TRPV Family in ccRCC

**Figure 6:**
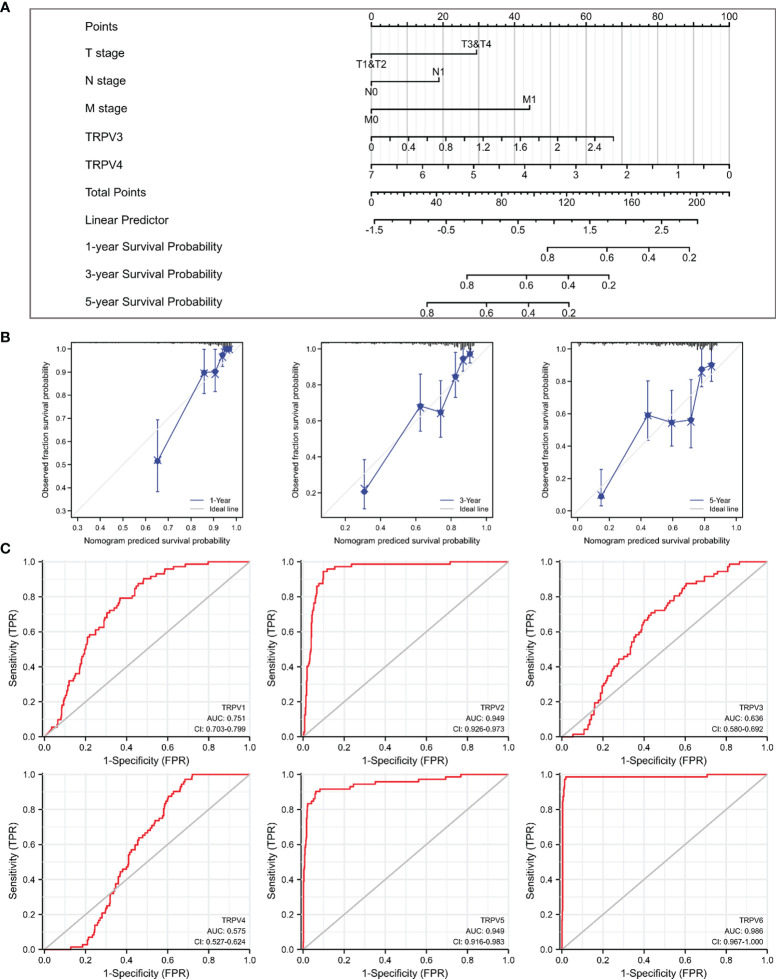
Prognostic and diagnosis values of TRPV family in ccRCC. **(A)** A nomogram model integrates TRPV3/4 expression and TNM stages for predicting the probability of 1-, 3- and 5-year overall survival for ccRCC. **(B)** The calibration curve of the nomogram model. **(C)** ROC analysis of TRPV family members. TRPV2/5/6 shows promising discrimination power between ccRCC and normal tissues.

Given that TRPV3 and TRPV4 were potential prognosis biomarkers, we developed a nomogram to predict the overall survival of ccRCC patients by fitting TRPV3/4 expression and TNM stage. The higher point on the Nomogram model, the worse the survival for ccRCC patients (**Figure 6A**). The calibration curve demonstrated that the predicted results were in good agreement with the observed results (**Figure 6B**). In summary, this nomogram may be a model for predicting overall survival in ccRCC combined TRPV3/4 mRNA expression with TNM stage than an individual prognostic factor. Afterwards, we evaluated the diagnostic value of TRPV family members by receiver operating characteristic (ROC) curve. As a result, the area under curve (AUC) of TRPV2/5/6 was 0.949, 0.949, and 0.986, respectively (**Figure 6C**). Due to the high performance discriminating ccRCC from the control samples (AUC>0.9), TRPV2/5/6 might potential diagnostic biomarkers for ccRCC patients.

### TRPV3 Expression and Clinical Parameters of ccRCC Patients

**Figure 7:**
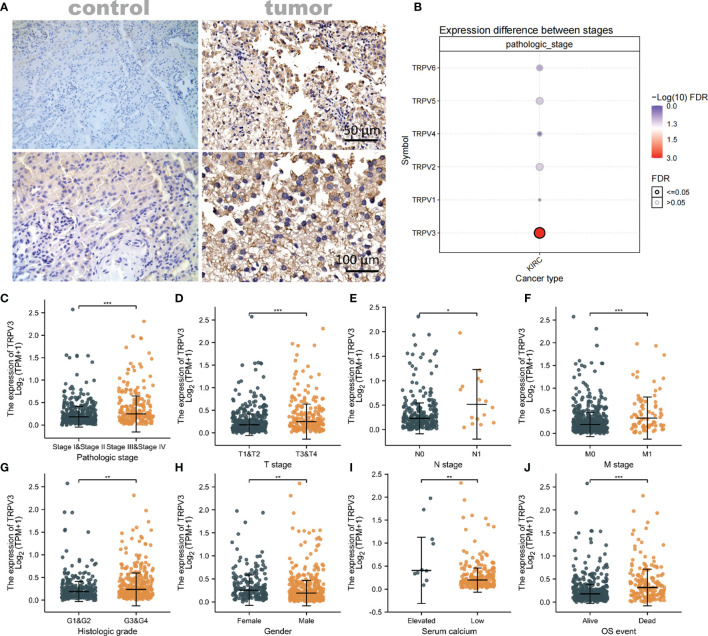
Association of TRPV3 mRNA expression with Clinical Parameters. **(A)** Representative images of TRPV3 expression in ccRCC tissues and normal controls. Original magnifications 50× and 100×. **(B)** Correlation between the TRPV1-6 expression and the pathologic stage of ccRCC patients. The mRNA expression of TRPV3 in ccRCC based on **(C)** Pathologic stage; **(D)** T stage; **(E)** N stage; **(F)** M stage; **(G)** Histologic stage; **(H)** Gender; **(I)** Serum calcium; **(J)** OS event. *P<0.05; **P<0.01; ***P<0.001.

Considering that TRPV3 plays a prominent role in ccRCC, the IHC staining was performed to validate the protein levels of TRPV3 expression between ccRCC tissues and normal tissues. As expected, an obviously increased protein level was observed in ccRCC tissues compared with the paired normal tissues (**Figure 7A**). By GSCA database, we next assessed the relationship between TRPV1-6 expression and pathologic stage of ccRCC patients, and we noticed that only TRPV3 was associated with advanced stage (**Figure 7B**). Compared with stage I/II/III groups, TRPV3 expression was obviously upregulated in stage III/IV groups (**Figure 7C**). Regarding TNM stage, TRPV3 transcription level was higher in ccRCC patients classified as T3&T4, N1 and M1 (**Figures 7D–F**). Similar results were also observed that the TRPV3 expression was significantly upregulated in the G3&G4 groups compared to G1&G2 groups (**Figure 7G**). In terms of gender, the TRPV3 mRNA expression was remarkably elevated in female patients compared with male patients (**Figure 7H**). Furthermore, higher expression of TRPV3 was observed in serum calcium-elevated patients compared to serum calcium-low patients (**Figure 7I**). In addition, the TRPV3 level was dramatically increased in dead groups compared with alive ccRCC patients (**Figure 7J**). Since the TRPV3 level was significantly different in subgroups with different clinical parameters, we further analyzed the correlation between TRPV3 expression and clinical variables by univariate logistic regression. As a result, high mRNA expression of TRPV3 was correlated with poor prognostic clinical parameters. Elevated TRPV3 expression in ccRCC was significantly correlated with high T stage (p<0.001), pathologic stage (p=0.001), histologic grade (p=0.022), and gender-female (p=0.012) (**Table 2**). These results implicated that TRPV3-high expression patients were prone to progress to a more advanced stage than TRPV3-low expression patients.

**Table 2:** Relationship between TRPV3 expression and clinicopathological characteristics (logistic regression).

Characteristics	Total(N)	Odds Ratio (OR)	P value
T stage (T3&T4 vs. T1&T2)	539	1.919 (1.342-2.756)	**<0.001**
N stage (N1 vs. N0)	257	2.648 (0.894-9.683)	0.100
M stage (M1 vs. M0)	506	1.589 (0.976-2.617)	0.065
Pathologic stage (Stage III&Stage IV vs. Stage I&Stage II)	536	1.803 (1.269-2.571)	**0.001**
Gender (Male vs. Female)	539	0.633 (0.442-0.905)	**0.012**
Age (>60 vs. <=60)	539	1.259 (0.898-1.767)	0.182
Histologic grade (G3&G4 vs. G1&G2)	531	1.490 (1.059-2.101)	**0.022**
Primary therapy outcome (CR vs. PD&SD&PR)	147	1.173 (0.441-3.336)	0.754
Serum calcium (Low vs. Elevated)	213	0.238 (0.035-0.978)	0.074

Bolded values are significant at p < 0.05.

### DNA Methylation Analysis of TRPV3 in ccRCC

**Figure 8:**
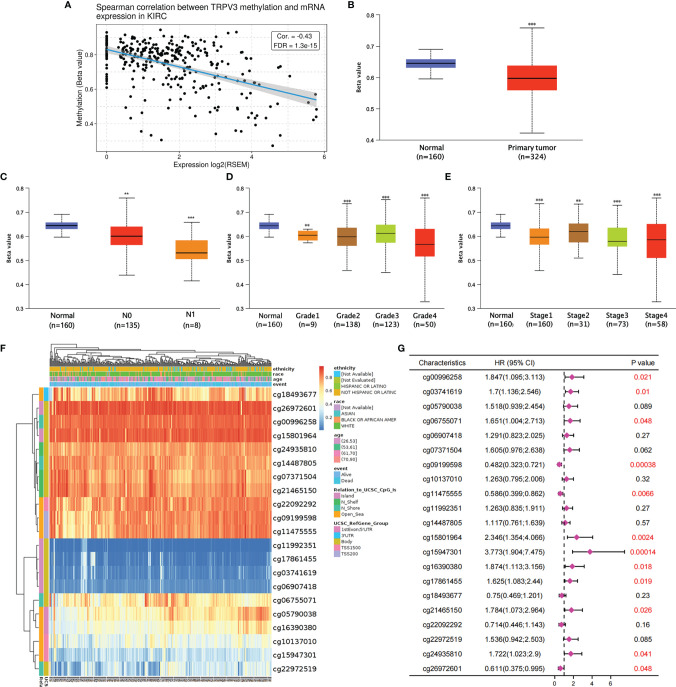
The DNA methylation analysis of TRPV3 in ccRCC. **(A)** Correlation between the TRPV3 mRNA expression and DNA methylation levels in ccRCC. **(B)** The promoter methylation level of TRPV3 in ccRCC tissues. The promoter methylation level of TRPV3 in ccRCC based on **(C)** nodal metastasis status; **(D)** tumor grade; **(E)** individual cancer stage. **P<0.01; ***P<0.001. **(F)** The heatmap of DNA methylation of TRPV3 in ccRCC obtained from MethSurv database. **(G)** The prognostic value of DNA methylation of TRPV3 in ccRCC with different CpG sites.

Subsequently, the GSCA tool was introduced to analyzed TRPV3 methylation and TRPV3 mRNA　expression. As shown in **Figure 8A**, TRPV3 expression was significantly negatively correlated with its methylation levels in ccRCC (r= -0.43, p < 0.001). Compared with normal samples, the DNA methylation levels of TRPV3 were dramatically lower in ccRCC tissues (**Figure 8B**). Furthermore, DNA methylation levels of TRPV3 was further reduced in patients with nodal metastasis (**Figure 8C**), advanced tumor grade (**Figure 8D**), and higher cancer stages (**Figure 8E**). Therefore, the decreased DNA methylation level of TRPV3 may be a potential index to reflect the clinical characteristics of patients with ccRCC. Next, we obtained the methylation map of TRPV3 from MethSurv database. According to the data, 21 CpG sites of TRPV3 were found (**Figure 8F**), and 12 CpGs of TRPV3 were associated with a significant prognosis, including cg00996258, cg03741619, cg06755071, cg09199598, cg11475555, cg15801964, cg15947301, cg16390380, cg17861455, cg21465150, cg24935810, and cg26972601 (**Figure 8G**). These results suggest that DNA methylation of TRPV3 might be involved in the development and progression of ccRCC, which was closely related to the prognosis of patients with ccRCC.

### Function Enrichment Analysis of TRPV3 in ccRCC

**Figure 9:**
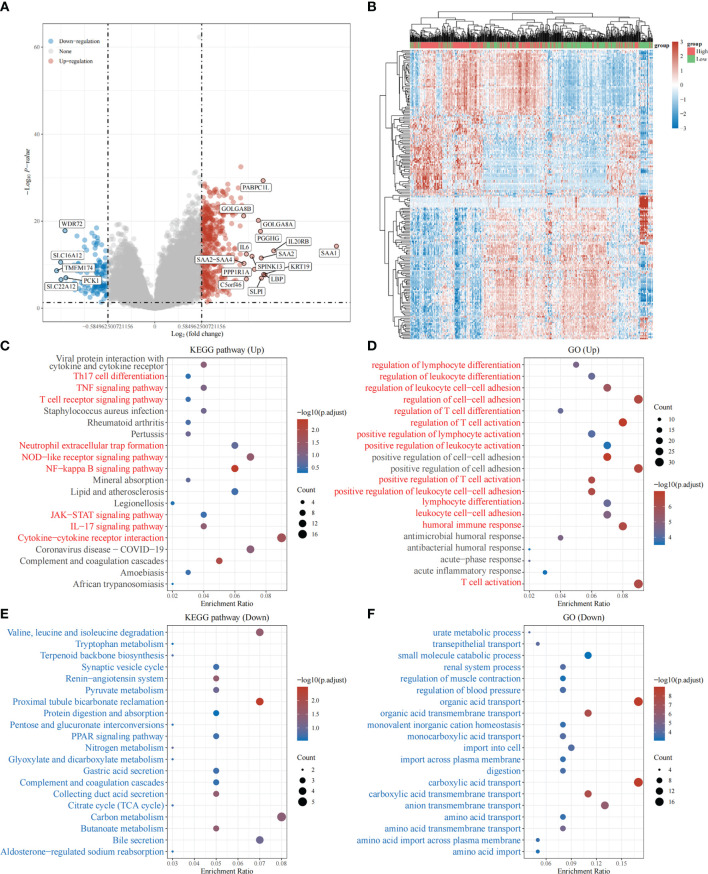
Function enrichment analysis of TRPV3-related gene sets in ccRCC. **(A)** Differentially expressed genes (DEGs) for high expression of TRPV3 vs low expression of TRPV3 in ccRCC were shown in the volcano plot, with orange dots representing significantly up-regulated genes and blue dots representing significantly down-regulated genes in ccRCC with high expression of TRPV3. **(B)** The heatmap exhibits the expression level. **(C)**Enrichment analysis for KEGG pathway of up-regulated genes. **(D)** Enrichment analysis for GO term of up-regulated genes. **(E)** Enrichment analysis for KEGG pathway of down-regulated genes. **(F)** Enrichment analysis for GO term of down-regulated genes.

Based on the above, we found that the expression levels of TRPV3 was significantly increased in ccRCC and high expression was related with a significantly worse survival. However, the changes of signaling pathway caused by increased TRPV3 expression in ccRCC is still unknown. To broaden the understanding of the functional implication of TRPV3 in ccRCC, the function enrichment analysis for TRPV3-related gene sets in ccRCC was performed. We first identified 537 differently expressed genes (DEGs) between TRPV3-high and TRPV3 low-expression groups using TCGA-KIRC cohort, including 420 up-regulated genes and 117 down-regulated genes in theTRPV3 high-expression group compared with the TRPV3 low-expression group (**Figures 9A, B**). KEGG analysis revealed up-regulated genes associated with TNF signaling pathway, T cell receptor signaling pathway, and Neutrophil extracellular trap formation (**Figure 9C**). GO term analysis revealed that the up-regulated genes were significantly enriched in immune-related pathways, such as regulation of lymphocyte differentiation, regulation of T cell activation, positive regulation of lymphocyte activation, and humoral immune response (**Figure 9D**). For down-regulated genes, KEGG analysis showed that these genes were involved in metabolism-related pathway, including Butanoate metabolism, Glyoxylate and dicarboxylate metabolism, and Pyruvate metabolism (**Figure 9E**). GO term analysis also demonstrated that the down-regulated genes were enriched in metabolism regulation, such as urate metabolic process, amino acid transport, and amino acid transmembrane transport (**Figure 9F**). These results implied that TRPV3 might be involved in the occurrence and development of ccRCC through metabolism and immune regulation.

### Immune Infiltration Analysis of TRPV3 in ccRCC

**Figure 10:**
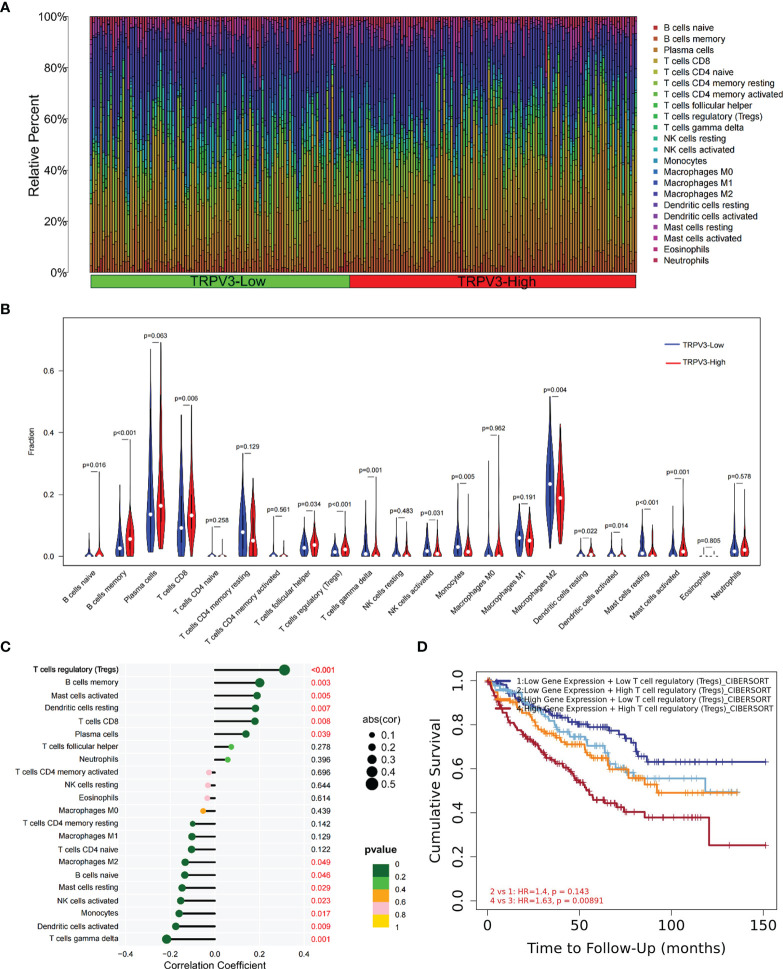
Immune cell infiltration analysis of TRPV3 in ccRCC. **(A)** Proportions of 22 tumor-infiltrating immune cells (TIICs) in ccRCC samples. **(B)** The difference of the levels of 22 TIICs between high and low TRPV3 expression groups. **(C)** The forest plot shows the relations between the abundance of 24 immune cells and TRPV3 mRNA expression. The transcription level of TRPV3 was significantly positively associated with the levels of Treg cells infiltration in ccRCC tissues. **(D)** Survival analysis of Treg cells infiltration in ccRCC patients according to high and low TRPV3 expression.

Given that TRPV3 might play a crucial regulatory role in the differentiation and activation of immune cells, we further explored the correlation between TRPV overexpression and tumor-infiltrating immune cells (TIICs) in ccRCC microenvironment. The histogram summarized the composition of 22 types of TIICs in 265 ccRCC samples based on GSE73731 datasets (**Figure 10A**). The differences in the proportion of each 22 TIICs between the TRPV3-low (n=132) and TRPV3-high (n=132) groups were further analyzed. Compared with TRPV3 low-expression groups, the ccRCC microenvironment with TRPV3-high expression contained a higher proportion of B cells memory, T cells CD8, T cells regulatory (Tregs), Mast cells activated, while T cells gamma delta, Monocytes, Macrophages M2, Mast cells resting infiltrated less (p<0.01; **Figure 10B**). Furthermore, we evaluated the correlation between TRPV3 expression and levels of 22 TIICs. Correlation analysis showed that TRPV3 expression was significant with the accumulation of several tumor-infiltrating immune cells, especially Treg cells (r=0.31, p<0.001; **Figure 10C**). Since TRPV3 expression had a significant correlation with infiltration levels of Treg cells, we investigated whether the poor prognosis of ccRCC patients mediated by Treg cells was affected by TRPV3 expression. As shown in **Figure 10D**, in TRPV3-high expression groups, increased levels of Treg cells contribute to poor clinical survival. However, there was no significant association between Treg cells levels and the prognosis of ccRCC patients in the groups with low expression TRPV3. These results supported the findings that TRPV3 might function as an important immunoregulatory factor in ccRCC.

### Relation Between TRPV3 With Immune Checkpoints

**Figure 11:**
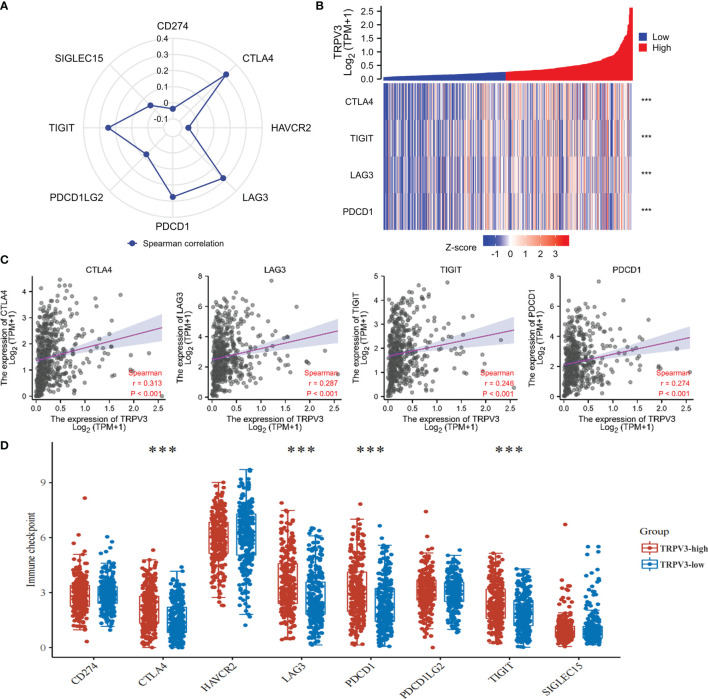
Correlation between the TRPV3 expression and immune checkpoints in ccRCC. **(A)** Radar chart evaluating the relationship of ACE2 and immune checkpoint molecules in ccRCC. **(B)** The co-expression heatmap of TRPV3 and the expression of CTLA4, PDCD1, TIGIT and LAG3. **(C)** Scatter diagrams showed that TRPV3 mRNA expression was significantly positively correlated with the expression of CTLA4, PDCD1, TIGIT and LAG3. **(D)** The expression of CTLA4, PDCD1, TIGIT and LAG3 was significantly upregulated in the TRPV3 high-expression group compared to the low-expression group. ***P<0.001.

The efficacy of immunotherapy not only needs adequate immune cells infiltrating in the ccRCC microenvironment but also depends on the sufficient expression of immune checkpoints. Consequently, we assessed the relationship of TRPV3 expression with immune checkpoints, including CTLA4, HAVCR2, LAG3, PDCD1, PDCD1LG2, TIGIT, SIGLEC15, and CD274. The results suggest that the TRPV3 level was significantly positively correlated with the expression of CTLA4, PDCD1, TIGIT, and LAG3 (**Figures 11A–C**). Furthermore, these immune checkpoints were expressed at significantly higher levels in TRPV3 high -xpression groups compared to TRPV3 low-expression groups (**Figure 11D**).

### Construction of a Potential SNHG3/AL513497.1-miR-10b-5p-TRPV3 Axis in ccRCC

**Figure 12:**
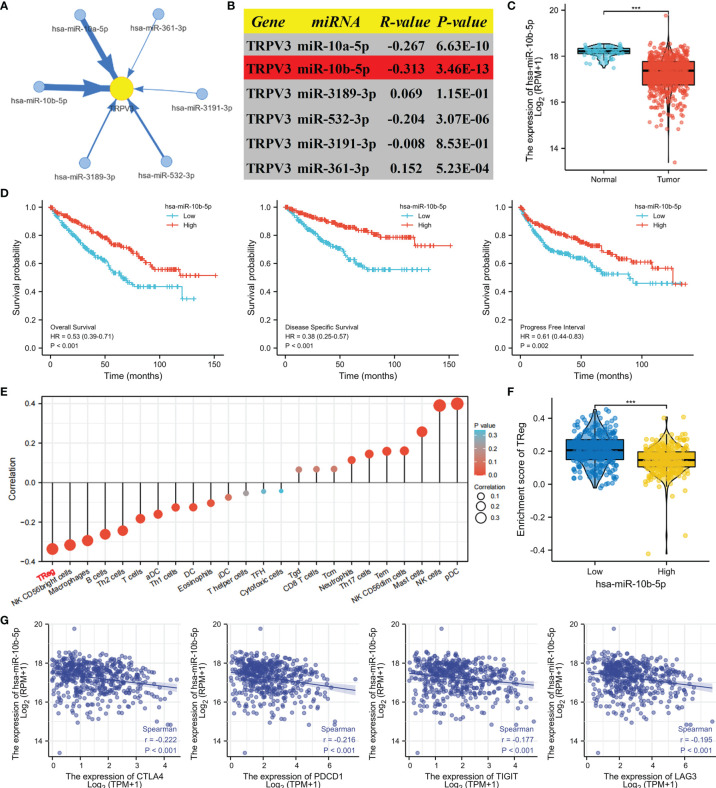
Identification of miR-10b-5p as a potential upstream miRNA of TRPV3 in ccRCC. **(A)** The miRNA-TRPV3 regulatory network analyzed by StarBase database. **(B)** The correlation between TRPV3 and candidate miRNAs. **(C)** miR-10b-5p was downregulated in ccRCC. ***P<0.001. **(D)** ccRCC patients had a better survival with high expression of miR-10b-5p. Identification of SNHG3 and AL513497.1 as potential upstream lncRNAs of miR-10b-5p in ccRCC. **(E)** The forest plot shows the relations between the abundance of 24 immune cells and miR-10b-5p expression. The expression of miR-10b-5p was significantly negatively associated with the levels of Treg cells infiltration in ccRCC tissues. **(F)** The level of Treg cell was significantly down-regulated in the miR-10b-5p high-expression group compared to the low-expression group. ***P<0.001. **(G)** Scatter diagrams showed that miR-10b-5p expression was significantly negatively correlated with the expression of CTLA4, PDCD1, TIGIT and LAG3.

Next, we attempted to uncover the upstream regulatory structure of TRPV3 in ccRCC cancer. Through GSCA database, six potential binding miRNAs of TRPV3 were obtained, involving miR-10a-5p, miR-10b-5p, miR-3189-3p, miR-532-3p, miR-3191-3p, miR-361-3p (**Figure 12A**). Among them, miR-10b-5p had the strongest correlation with TRPV3 (r=-0.313; **Figure 12B**). Besides, miR-10b-5p was significantly downregulated in ccRCC (**Figure 12C**), and its low expression associated with worse survival (**Figure 12D**). These findings suggest that miR-10b-5p might have the most potential regulatory miRNA of TRPV3 in ccRCC. Considering that TRPV3 expression was significantly positively associated with Treg cells infiltration and immune checkpoints expression, we analyzed the correlation between miR-10b-5p expression and these immune signatures. Conversely, miR-10b-5p was dramatically correlated with Treg cells infiltration (r=−0.337, P < 0.001; **Figure 12E**). Compared with miR-10b-5p high-expression groups, the ccRCC microenvironment with miR-10b-5p low expression contained a higher proportion of Treg cells (**Figure 12F**). Besides, the expression of miR-10b-5p was significantly negatively correlated with immune checkpoints (CTLA4, PDCD1, TIGIT, and LAG3) (**Figure 12G**).

**Figure 13:**
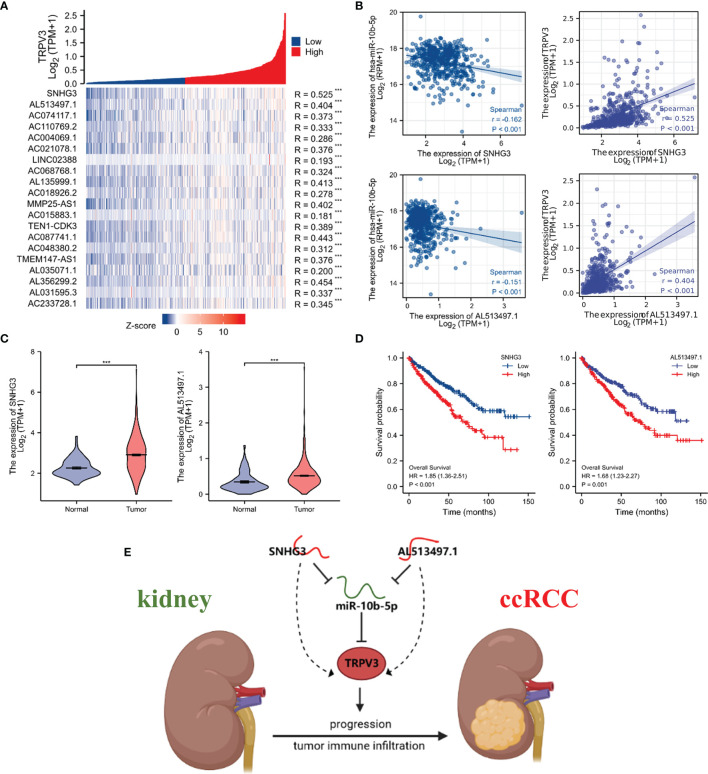
Identification of SNHG3 and AL513497.1 as potential upstream lncRNAs of miR-10b-5p in ccRCC. Correlation analysis of potential lncRNAs in ccRCC. **(A)** Co-expression heatmap showed that potential lncRNAs were positively correlated with TRPV3. **(B)** SNHG3 and AL513497.1 were negatively correlated with miR-10b-5p while positively correlated with TRPV3. **(C)** SNHG3 and AL513497.1 were upregulated in ccRCC. ***P<0.001. **(D)** High expression of SNHG3 and AL513497.1 predicted a worse survival of ccRCC patients. **(E)** The model of SNHG3/AL513497.1-miR-10b-5p-TRPV3 axis in carcinogenesis of ccRCC.

We subsequently predicted the upstream lncRNAs of miR-10b-5p. In total, 89 potential lncRNAs regulating miR-10b-5p were predicted by starBase database. Survival analysis was performed for each lncRNAs and screened out 23 lncRNAs whose high expression were associated with poor prognosis (**Figure S2**). Among them, 20 potential lncRNAs highly expressed in ccRCC tissues compared with control samples (**Figure S3**). According to the competing endogenous RNA (ceRNA) hypothesis, lncRNA could increase mRNA expression by competitively binding to shared miRNAs. Therefore, there should be negative correlation between lncRNA and miRNA or positive correlation between lncRNA and mRNA. Thus, the correlation analysis was performed, and the results revealed that all 20 potential lncRNAs were positively correlated with TRPV3 expression (**Figure 13A**) whereas only SNHG3 and AL513497.1 were significantly negatively correlated with miR-10b-5p (**Figure 13B**). Taking expression analysis, survival analysis, and correlation analysis into consideration, SNHG3 and AL513497.1 were identified as having the most potential upstream lncRNAs of miR-10b-5p (**Figure 13**). Finally, we drew the predicted model of the SNHG3/AL513497.1-miR-10b-5p-TRPV3 axis linked to the prognosis and development of ccRCC (**Figure 13E**).

## Discussion

As the main calcium channel protein, TRPV family have an impact on the biological behavior of various tumors based on its regulation of regulating calcium ion influx (28). The dysregulation of TRPVs expression may change the cellular membrane Ca2+ dependent response of living tissues, and thus influence the expression of corresponding genes to promote or inhibit cancer (29). A previous study reported that the hepatocyte growth factor promoted the migration of human hepatoblastoma cells by stimulating the activity of TRPV1, increasing intracellular calcium and triggering the signal cascade (30). Another study revealed that TRPV4 might regulate cell softness and extracellular matrix protein expression through the Ca 2+ dependent AKT-E-cadherin signal axis, thus regulating metastasis of breast cancer (31). However, few studies have explored the role of TRPVs in ccRCC, and the prognosis and biological significance of TRPV family members in ccRCC had not yet been elucidated. For the first time, we comprehensively analyzed the expression, mutation, diagnosis and prognosis value, DNA methylation, ceRNA network, and associations with immune cell infiltration and immune checkpoints of TRPV family in ccRCC.

First, we focused on the expression and clinical significance of TRPVs in ccRCC. Expression analysis showed that TRPV2/3 was highly expressed in ccRCC tissues compared with normal kidney tissues both in the TCGA-KIRC dataset and the GSE53757 dataset. However, TRPV5/6 was found to be downregulated in ccRCC tissues, which is consistent with the results of previous studies (15). The expression or activity of TRPV channels is deregulated along with the tumor progression, but the mutation of TRPV family genes is not common (32). The genetic alterations of TRPV family members in ccRCC was also assessed, and the results showed that TRPVs were rarely mutated (nearly 7 frequencies), and the alterations in the TRPV family had no influence on the survival of ccRCC patients. Therefore, our findings also demonstrate that TRPV family was highly conserved, and dysregulation of the TRPV family members in ccRCC was not attributed to gene mutations. Multiple studies have identified TRPVs as promising biomarkers of tumor diagnosis, prognosis, and progression. For example, increased expression of TRPV1 predicted the prolonged survival of patients with liver cancer (33). An integrated analysis reported that TRPV1 is a novel tumor suppressor and prognosis marker for ccRCC. High expression levels of TRPV1 predicted a favorable overall survival and disease-free survival of 1, 3, 5, and 10 years in patients with ccRCC (17). In gastric adenocarcinoma tissues, the protein level of TRPV4 is increased, and the higher TRPV4 expression, the deeper the tumor invasion and lymph node metastasis, and the higher the TNM stage (34). Another research reported that TRPV4 expression in keratinocytes of human non-melanoma skin cancer is significantly reduced, and the TRPV4 level is associated with IL-18 secretion, which indicated that TRPV4 might serve as a biomarker for the early diagnosis of skin cancer (35). Thus, we evaluated the diagnosis and prognosis power of TRPV family members in ccRCC. The ROC analysis revealed that TRPV2/5/6 had a high performance for distinguishing ccRCC patients from healthy individuals (AUC>0.9), suggesting that TRPV2/5/6 might serve as a diagnostic biomarker for patients with ccRCC. Furthermore, survival analysis showed that high expression of TRPV3 was associated with poor prognosis of ccRCC patients, whereas high expression of TRPV4 conferred a favorable prognosis. These findings implied that TRPV3 and TRPV4 are potential prognosis biomarkers for ccRCC patients.

Due to the different structure and function of each subtype, TRPV family members show different or even opposite roles in the regulation of tumor (36). In human hepatocellular carcinoma (HCC), TRPV2 expression was found in increased levels at both mRNA and protein levels, and its high expression inhibited tumor differentiation (37). However, the expression levels of TRPV1 in HCC was decreased, and high expression of TRPV1 was associated with a better prognosis with HCC patients (33). To sum up, distinct TRPV channels might display clearly different roles in a cancer-type-dependent manner. In the present study, we found that TRPV3 was highly expressed in ccRCC tissues and correlated with worse survival of ccRCC tissues. However, ccRCC patients with high expression of TRPV4 experienced a prolonged survival. The opposite role of TRPV3 and TRPV4 in ccRCC needs to be further verified in more experiments. Considering the potential oncogenic role of TRPV3 in ccRCC, the relationship of TRPV3 expression with clinical parameters of ccRCC patients was analyzed, and the results showed that the TRPV3 level was higher in the late-stage and high-grade ccRCC patients. DNA methylation is an important epigenetic alteration in the process of tumorigenesis development (38). Genome-wide hypomethylation is a common phenomenon in human tumors. Hypomethylation usually occurs on the promoter of proto-oncogenes, which activates and induces cell carcinogenesis (39, 40). In our study, we found that TRPV3 mRNA expression was negatively correlated with DNA methylation levels. Besides, the promoter methylation level of TRPV3 was significantly lower in ccRCC tissues compared to normal tissues, and the methylation level was further reduced with tumor progression and metastasis progress. According to the methylation map, 12 CpGs of TRPV3 were associated with a significant prognosis. These results imply that the pattern of methylation changes of TRPV3 may be one of the reasons for the upregulation of TRPV3 expression in ccRCC, and is involved in the cancer initiation and progression.

In order to elucidate the biological role of TRPV3 in ccRCC, we identified DEGs according to TRPV3 high- and low- expression, and functional enrichment analysis was performed. The up-regulated genes mediated by TRPV3 overexpression were found to be involved in the differentiation and activation of immune cells. Indeed, a previous study had reported that the expression of TRPV channels increased during activation of T cells, and TRPV channels could regulate mitogenic-mediated T cell activation and effector cytokines production (41). These findings suggest that TRPV3 might play complex immune regulatory roles in the ccRCC microenvironment. Therefore, we evaluated the effect of TRPV3 expression on the proportion of tumor-infiltrating immune cells (TIICs) in ccRCC. As a result, the proportions of T cells regulatory, B cells memory, T cells CD8, and Mast cells activated significantly increased in the high TRPV3 expression group, whereas those of T cells gamma delta, Monocytes, Macrophages M2, Mast cells were significantly increased in the low TRPV3 expression groups. Furthermore, correlation analysis revealed that TRPV3 significantly correlated with the accumulation of these TIICs, especially Treg cells. It is well known that Treg cells are subsets of T cells with negative regulatory functions and play an important role in maintaining immune homeostasis (42). Treg cells are not found in normal renal tissue, but in the ccRCC microenvironment, they are enriched and infiltrated around the tumor cells, inhibiting the activation and function of effector T cells and promoting tumor immune escape (43). In general, high levels of tumor-infiltrating Treg cells correlated with poor survival outcomes in patients with ccRCC (44). Furthermore, a study by Zheng et al. found that the TRPV1 level was inversely related to the expression of Foxp3 (Treg marker) in ccRCC microenvironment (r = -0.302, P = 3.23e-11) (17). In our study, we found that increased levels of Treg cells contribute to the poor prognosis of ccRCC patients with high TRPV3 expression. However, in patients with low TRPV3 expression, the levels of Treg cells had no significant impact on ccRCC prognosis. Taken together, TRPV3 was a potential immunomodulatory factor in ccRCC, and targeting TRPV3 might inhibit the immunosuppressive effect of Treg cells and increase the efficacy of immunotherapy.

Studies have found that lncRNA can play a sponge role on miRNA; act as ceRNA to competitively bind miRNA; affect the combination of miRNA and mRNA; and thereby regulate the occurrence, development, and metastasis of tumors (45). The regulatory network of LncRNA­ miRNA­ mRNA exists widely in cancer, affecting the expression of tumor-related genes and playing an important biological role (46). Therefore, we attempt to construct a ceRNA regulatory network of TRPV3 in ccRCC through bioinformatics analysis. Firstly, we obtained six potential upstream miRNAs of TRPV3, and miR-10b-5p was identified as the highest potential upstream miRNA of TRPV3 through correlation analysis, expression analysis, and survival analysis. Previous studies have reported that miR-10b-5p played an important anti-tumor role in ccRCC by modulating proliferation, migration, and apoptosis of tumor cells (47). Consistently, our study also confirmed that miR-10b-5p was low- expressed in ccRCC and loss expression of miR-92a-3p led to poor prognosis in ccRCC patients. Additionally, miR-10b-5p was significantly negatively correlated with TRPV3, suggesting that miR-92a-3p might play inhibitory roles in ccRCC by targeting TRPV3. Next, we predicted the upstream LncRNAs of miR-10b-5p in ccRCC, and 78 candidate lncRNAs were preliminarily selected from starBase database. By a combination of survival analysis, expression analysis, and correlation analysis, SNHG3 and AL513497.1 were identified as the most promising lncRNAs of miR-10b-5p in ccRCC. According to the ceRNA mechanism, the potential lncRNAs of miR-10b-5p-TRPV3 axis should be oncogenic lncRNAs in ccRCC. Our study showed that the expression of SNHG3 and AL513497.1 was increased in ccRCC, and associated with worse survival of ccRCC patients. A previous study also demonstrated that SNHG3 overexpression promoted tumor proliferation and migration in ccRCC (48). In addition, we found that SNHG3 and AL513497.1 were positively correlated with TRPV3 while negatively correlated with miR-10b-5p. Conclusively, we developed a potential SNHG3/AL513497.1-miR-10b-5p-TRPV3 axis in the progress of ccRCC, which might provide effective prognostic biomarkers and promising treatment targets for ccRCC.

To sum up, TRPV2/3 expression was upregulated while TRPV5/6 expression was downregulated in ccRCC tumor tissues. TRPV2/5/6 was a potential diagnostic biomarker and TRPV3/4 was a potential prognosis biomarker for patients with ccRCC. Besides, aberrant TRPV3 expression in ccRCC may be caused by DNA methylation dysregulation. In addition, TRPV3 has a significantly positive correlation with Treg cells infiltration and immune checkpoints expression. Activating or blocking TRPV3-related signaling pathways may provide novel insights for immunotherapy of ccRCC patients.

## Data Availability Statement

The datasets presented in this study can be found in online repositories. The names of the repository/repositories and accession number(s) can be found in the article/**Supplementary Material**.

## Ethics Statement

The studies involving human participants were reviewed and approved by Institutional Research Ethics Committee of Tongji University. The patients/participants provided their written informed consent to participate in this study. Written informed consent was obtained from the individual(s) for the publication of any potentially identifiable images or data included in this article.

## Author Contributions

YJ and XS: Conceptualization. YJ, YZ, and DH: methodology. CZ and DH: software. CZ, YZ, and XS: validation. YJ: formal analysis and investigation. YJ: writing-original draft preparation and visualization. XS and WG: writing-review and editing, supervision and project administration. All authors have read the final version of this manuscript.

## Funding

This study was supported by the National Natural Science Foundation of China (81800089), and a research grant from Shanghai Tenth People’s Hospital to WG (2021SYPDRC033).

## Conflict of Interest

The authors declare that the research was conducted in the absence of any commercial or financial relationships that could be construed as a potential conflict of interest.

## Publisher’s Note

All claims expressed in this article are solely those of the authors and do not necessarily represent those of their affiliated organizations, or those of the publisher, the editors and the reviewers. Any product that may be evaluated in this article, or claim that may be made by its manufacturer, is not guaranteed or endorsed by the publisher.
