# Supplementary material for: Multi-Omics Analysis of the Prognosis and Biological Function for TRPV Channel Family in Clear Cell Renal Cell Carcinoma
Source: Front Immunol. 2022 Apr 26;13:872170. doi: 10.3389/fimmu.2022.872170 (PMC9086597; doi:10.3389/fimmu.2022.872170)
Supplement: Supplementary file 4 [file DataSheet_4.docx]

Supplementary Material

## Supplementary Figure

##
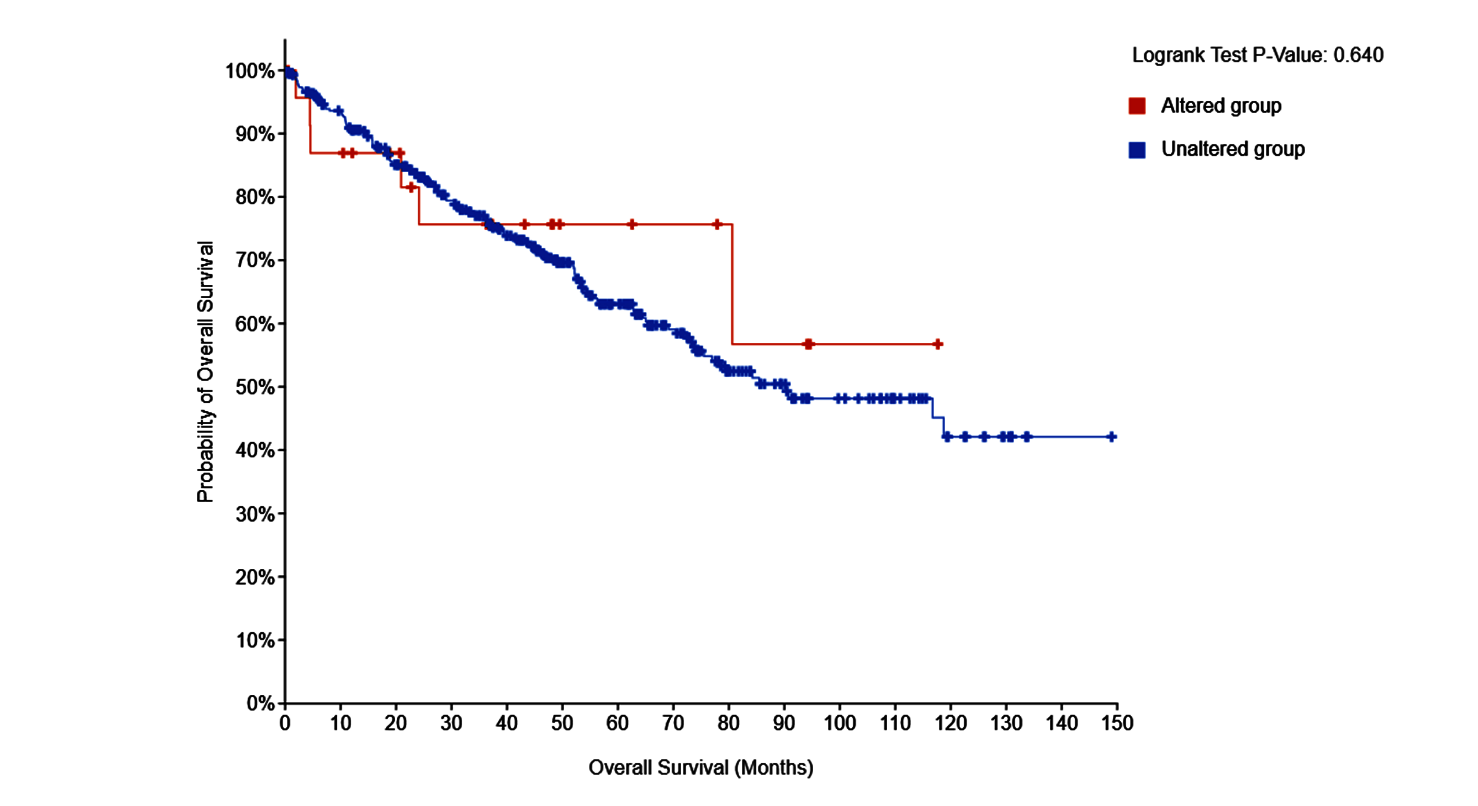
Figure S1. Relationship between TRPV family mutations and prognosis in patients with ccRCC. TRPV family mutations had no statistical significance in predicting the prognosis of patients with ccRCC.


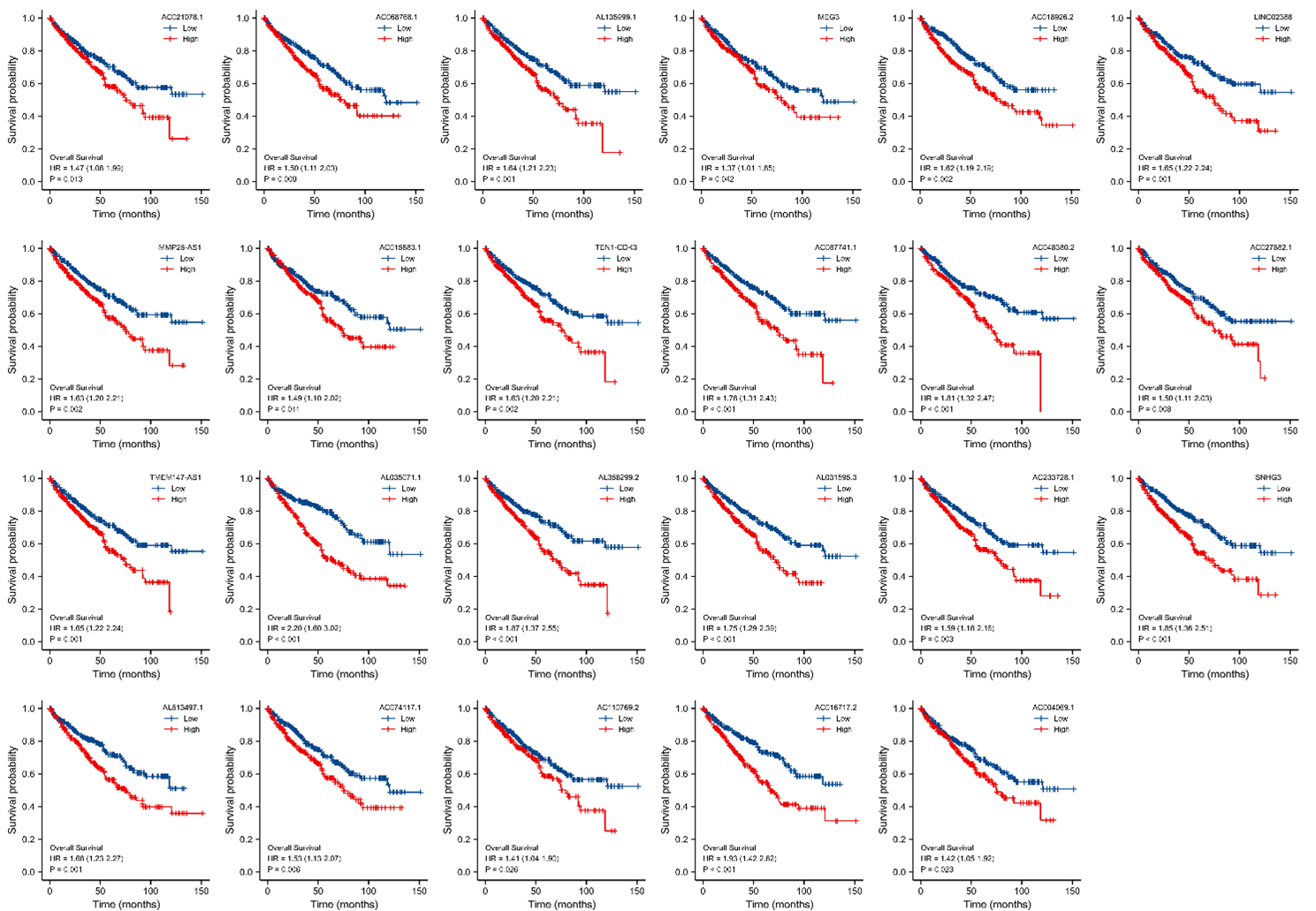


**Figure S2.** Survival analysis of potential lncRNAs in ccRCC.


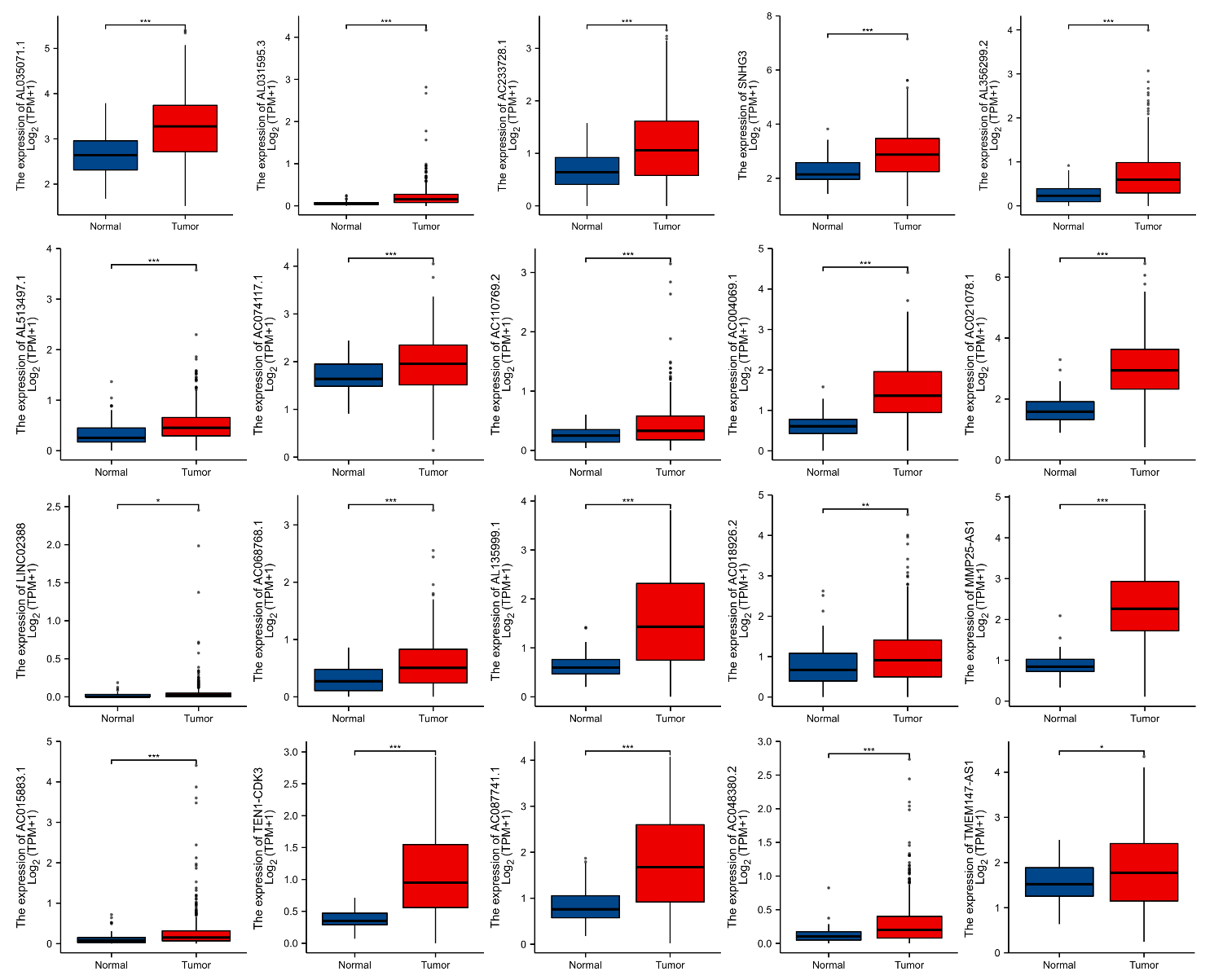


**Figure S3.** Expression analysis of potential lncRNAs in ccRCC.
